# Gross and Fine Dissection of Inner Ear Sensory Epithelia in Adult Zebrafish (*Danio rerio*)

**DOI:** 10.3791/1211

**Published:** 2009-05-08

**Authors:** Jin Liang, Shawn M. Burgess

**Affiliations:** Genome Technology Branch, National Human Genome Research Institute; Neuroscience and Cognitive Science Program, University of Maryland

## Abstract

Neurosensory epithelia in the inner ear are the crucial structures for hearing and balance functions. Therefore, it is important to understand the cellular and molecular features of the epithelia, which are mainly composed of two types of cells: hair cells (HCs) and supporting cells (SCs). Here we choose to study the inner ear sensory epithelia in adult zebrafish not only because the epithelial structures are highly conserved in all vertebrates studied, but also because the adult zebrafish is able to regenerate HCs, an ability that mammals lose shortly after birth. We use the inner ear of adult zebrafish as a model system to study the mechanisms of inner ear HC regeneration in adult vertebrates that could be helpful for clinical therapy of hearing/balance deficits in human as a result of HC loss.

Here we demonstrate how to do gross and fine dissections of inner ear sensory epithelia in adult zebrafish. The gross dissection removes the tissues surrounding the inner ear and is helpful for preparing tissue sections, which allows us to examine the detailed structure of the sensory epithelia. The fine dissection cleans up the non-sensory-epithelial tissues of each individual epithelium and enables us to examine the heterogeneity of the whole epithelium easily in whole-mount epithelial samples.

**Figure:**
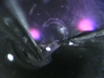


## Protocol

### Part 1: Preparation of the tissue

Euthanize the adult zebrafish with buffered MS-222 (Ethyl 3-aminobenzoate methanesulfonate salt, 0.03%).
Decapitate the fish. Cut off the lower jaw, place the head on the dissection pad with the ventral side up, and immobilize the tissue with insect pins. Remove the soft tissue ventral to the skull and crack open the ventral part of the skull capsule with a pair of fine tweezers under a dissection microscope.
Fix the fish head in paraformaldehyde (4%) at 4°C overnight.
Rinse the fixed fish head in PBS (Phosphate Buffered Saline, 1X) for 3 times, 5 minutes each time.

### Part 2: Gross dissection of the inner ear

Place the fish head on the dissection pad with the ventral side up. Immobilize it with insect pins. All following steps are carried out under the dissection microscope.
With a pair of fine tweezers, remove the skull bone over the inner ear and then carefully pull out the inner ear tissues: saccules, lagenae, and utricles. The saccule and lagena are tightly attach to each other and thus pull out together while the utricle (anterior to the saccule and lagena), will very likely to be detached from the other end organs during dissection.
Put the inner ear tissues in PBS (1X). If no further fine dissection is needed, rinse the tissue 2-3 times with PBS (1X) and it can be used for other experiments, e.g. cryosections.

### Part 3: Fine dissection of the inner ear sensory epithelia

The fine dissection can be carried out in a drop of PBS (1X) on a clean glass slide.
Remove the utricular otolith from the utricle with two pairs of fine tweezers, and, if desired, trim off the non-sensory epithelial tissue and the innervation from the epithelia with a pair of fine tweezers and a needle blade.
Remove the lagenar otolith before separating saccular and lagenar epithelia while try to keep the whole saccule as intact as possible. Place the tissue in the PBS drop with ventral side up. Use the needle blade to carefully cut in between the saccule and lagena. Trim off the excessive non-sensory epithelial tissue and the innvervation with fine tweezers and the needle blade, if desired.

### Part 4: Fluorescent staining of the hair cells in the sensory epithelia

Rinse the sensory epithelia with PBT (1XPBS and 1% Triton X-100) for 3 times, 5 min each time.
Incubate the epithelia with fluophore-labeled phalloidin (e.g. Alexa Flour 488 Phalloidin, 1:1,000 dilution) for 30 min at room temperature.
Rinse the sensory epithelia with PBT for 3 times, 10 min each time.
Mount the epithelia onto a slide with mounting medium and check the stained tissue under the fluorescent microscope with filters of the proper wavelength.
After successful dissection and staining, intact epithelia with strong hair cell bundle staining can be seen under the microscope (Fig. 1).

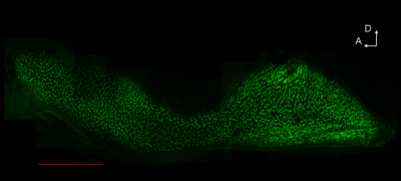
 **Figure 1. Hair cells of the saccular sensory epithelium were stained with Alexa Flour 488 Phalloidin (Green).** The tissue sample was dissected and stained as mentioned in the paper. Several pictures were taken at different positions of the sample and tiled together with Adobe Photoshop 7.0.  Scale bar = 150µm.

## Discussion

In this protocol, we described the gross and fine dissection of the inner ear sensory epithelia of adult zebrafish, which enables us to prepare tissue sample for studying the cellular and subcellular features of the sensory epithelia.

In order to carry out a successful dissection, the very first step, fixation, is very important. Make sure that you always use freshly prepared paraformaldehyde solution because old solution will result in under-fixation of the tissues that are hard to handle during the dissection. However, make sure that you don’t over-fix the tissues by leaving them in the fixation solution for too long, because it will interfere with the results of the immune/histological staining after the dissection. In addition, using well-maintained dissection tools that are in appropriate sizes is critical as well, especially for the fine dissection.

This gross dissection protocol can also be adapted for acquiring inner ear tissues for extraction of tissue-specific DNAs/RNAs/proteins. In that case, the dissection should be carried out right after the sacrifice of the fish without paraformaldehyde fixation. In addition, certain routine methods can be used to protect the degradation of the target macromolecules. For example, the fish head (lower-jaw removed) can be rinsed and then immersed in the RNAlater solution (Ambion) during the dissection for tissue-specific RNA extraction.

